# Nitrogen availability alters flavonoid accumulation in *Cyclocarya paliurus* via the effects on the internal carbon/nitrogen balance

**DOI:** 10.1038/s41598-019-38837-8

**Published:** 2019-02-20

**Authors:** Bo Deng, Yuanyuan Li, Dandan Xu, Qingqing Ye, Guihua Liu

**Affiliations:** School of Forestry and Landscape Architecture, Anhui Agricultural University, Hefei, Anhui 230036 China

## Abstract

*Cyclocarya paliurus* has traditionally been used in medicines and nutraceutical foods. The aims of this study were to determine whether flavonoid accumulation in *C. paliurus* is dependent on nitrogen (N) availability and to investigate the internal C (carbon)/N balance under controlled conditions. One-year-old seedlings were grown under five increasing available N level treatments (N1–5) and were harvested throughout the 15-d experimental period. The greatest total chlorophyll amount and photosynthetic rate were achieved during the intermediate N treatments (N3 and N4). The greatest starch level was detected in N3. The total C level was relatively stable, but the total N and free amino acid levels increased, which resulted in a decreased C:N ratio. The flavonoid contents in roots and stalks decreased, while leaves showed a different pattern (peaking in N3). The flavonoid level was closely correlated with flavanone-3-hydroxylase activity, which displayed a similar variation pattern, and their levels were significantly positively correlated with those of total C and starch. Thus, the partitioning of C among primary and secondary metabolisms could be responsible for flavonoid biosynthesis and provide the basis for maintaining high yields, which increases the nutritional values of crops and medicinal plants.

## Introduction

Secondary metabolites, like flavonoids, have special roles in determining plants quality because they contribute to the colours and flavours of vegetables and fruits, and may have high antioxidant levels that help protect humans from degenerative diseases^1–3^. Fertilization can increase biomass production in medicinal plant, vegetable and crop cultivation systems; however, it can decrease the biosynthesis and accumulation of physiologically active substances, including flavonoids and terpenes, and further impact the quality of raw materials^4,5^. As a crucial plant macronutrient that has potential trade-off effects between growth and the secondary metabolism rate, nitrogen (N) has been extensively studied^6^. Information on the effects of N levels have been acquired using different experimental systems, such as light- and nutrient-controlled tests on two freshwater macrophytes^7^, feeding different N forms to chamomile plants^6^, and the gene expression analysis of the flavonoid pathway^8^.

The biosynthesis of flavonoids, carbon (C)-based secondary substances, is greatly influenced by the N status of the plant, but there is still not a full understanding of the significance of regulating the effects of N. Increasing our knowledge is important not only to clarify the relationship between flavonoid biosynthesis and N metabolism, but also to increase the quality and yield of medicinal plants and crops. Based on the C–nutrient balance hypothesis, fertilization decreases the C:N ratios of plants, reducing surplus C production and decreasing the C-based defences while increasing the utilisation of assimilated N for defence^9^. The C–nutrient balance hypothesis produces a pattern similar to that predicted by the growth–differentiation balance hypothesis, which predicts that secondary metabolites tend to accumulate under intermediate resource levels owing to the excess pool of assimilates and that the defences can be produced relatively inexpensively^9,10^. These hypotheses partially form the theoretical basis of quality control in the cultivation of medicinal plants, but they have only partially been supported by studies.

**Figure 1:**
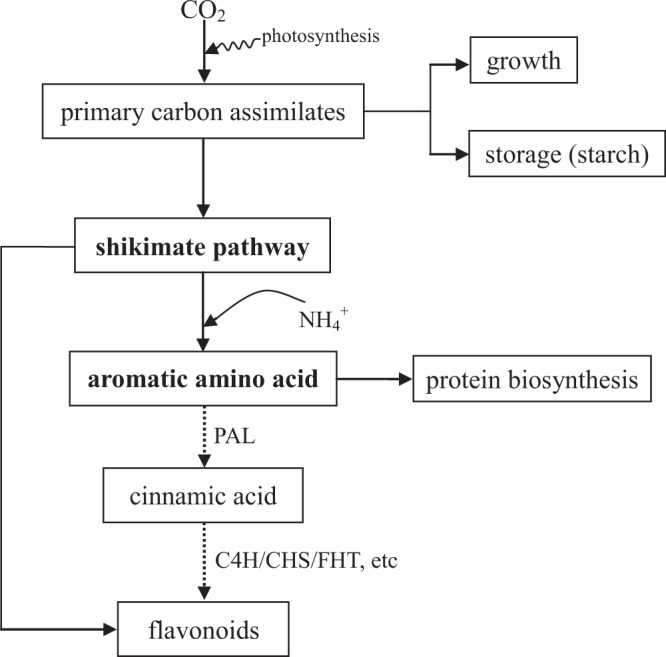
Correlation between flavonoid and amino acid synthesis in *Cyclocarya paliurus*. The solid and dotted lines indicate primary and secondary metabolism, respectively. PAL, phenylalanine ammonia lyase; C4H, cinnamate 4-hydroxylase; CHS, chalcone synthase; FHT, flavanone 3-hydroxylase.

In plants, the main biosynthesis flavonoid pathway is the shikimate pathway, which provides phenylalanine not only for amino acid and protein synthesis but also for the production of secondary metabolites, like flavonoids and terpenes^11^ (Fig. 1). Therefore, primary and secondary metabolism may compete for the available photosynthetic assimilates, and there is a trade-off in the C allocation^10^. Generally, plant photosynthesis is less sensitive to nutrient limitations (e.g. N) than growth, which implies that carbohydrate accumulations can exceed growth demands, resulting in their availability for conversion into secondary metabolites. In high available-nutrient environments, large amounts of carbohydrates are allocated to primary metabolism (protein synthesis), while secondary metabolism is limited. Overall, N may regulate the biosynthesis of flavonoids by controlling the C flow allocation between primary and secondary metabolism. This hypothesis has been partially confirmed. For example, high N fertilisation decreases flavonoid accumulation in plants^12,13^, and N shortage induces carbohydrate (such as starch and fructose) accumulation but decreases amino acid levels within plants^14,15^.

*Cyclocarya paliurus*, which is a deciduous tree that is widely distributed in the sub-tropical regions of China, belongs to the Juglandaceae family^16^. As a valuable medicinal woody tree species, the leaves of *C. paliurus* have long been used in traditional Chinese medicines and as a food resource^17^. Extracts from *C. paliurus* leaves have strong health-promoting effects, such as inhibiting protein tyrosine phosphatase 1B, improving mental efficiency and enhancing antihypertensive actions^18,19^. Chemical constituent studies have shown the presence of abundant physiologically active compounds, such as flavonoids and triterpenoids, in *C. paliurus*^19,20^. N fertilization can decrease the flavonoid accumulations in *Cyclocarya paliurus* leaves^21^. However, there is limited knowledge of the regulatory mechanisms involving N.

To obtain the more detailed information about the effects of N on flavonoid biosynthesis in *C. paliurus*, we investigated flavonoid accumulation and related primary metabolism processes. This study aims at illuminate the metabolic mechanism of N control in photosynthetic C allocation. The information will be of great value for increasing the health-promoting effects and establishing the optimal cropping strategies of *C. paliurus* plants.

## Materials and Methods

### Plant material and experimental design

Seeds of *C. paliurus* were collected from natural forests (a selected single tree) of Anji (Zhejiang, China) in late October, 2014. The collected seeds were firstly subjected to chemical scarification, followed by exogenous gibberellin A3 (GA3) treatments, and then stratification treatments using a method described by Fang *et al*.^22^. After stratification treatment for 3 months, the germinated seeds were firstly sown in plastic containers (5 cm in diameter, 15 cm in height) and then transplanted to the field (Baima, Nanjing, China) when the seedlings were about 6 cm in height.

In March 2016, the 1-year old plants were retransplanted to experimental containers (20 cm in diameter and 26 cm in height, 1:1 sand-perlite of planting substrate) in Hefei (Anhui, China) and grown in a controlled environment phytotron with 620 μmol·m^−2^·s^−1^ light intensity, a 12 h photoperiod, a 25 °C/15 °C diurnal/night temperature and a constant relative humidity of 65%. All plants were watered 2-days intervals with complete nutrient medium (160 mg/L NH_4_NO_3_, 408 mg/L KH_2_PO_4_, 136 mg/L CaSO_4_, 120 mg/L MgSO_4_, 8.4 mg/L EDTA-Fe (ethylenediaminetetraacetic acid), 3.8 mg/L KCl, 1.6 mg/L H_3_BO_3_, 0.3 mg/L MnSO_4_, 0.3 mg/L ZnSO_4_, 0.1 mg/L CuSO_4_, 0.1 mg/L Na_2_MoO_4_). After 3-week planting, fertilization treatment were conducted. Five fertilization levels were included in this study: 0 mM NH_4_NO_3_ (N1), 0.19 mM NH_4_NO_3_ (N2), 0.63 mM NH_4_NO_3_ (N3), 1.13 mM NH_4_NO_3_ (N4), 2.00 mM NH_4_NO_3_ (N5). These plants were irrigated 2-days intervals with different N-concentration solution from then on. Three replications were included in each treatment, and each replication consisted of 5 seedlings. Plants materials were harvested throughout the 15 d experiment.

### Measurements of photosynthesis and chlorophyll

The net photosynthesis rate was measured during the 9:00–11:00 under the photoperiod condition over the 15-day experiment. Three seedlings per replication were selected (the fully developed leaflets on the 5-th compound leaf below the apex of the plant) for measurements, using LI-6400XT photosynthetic system (LI-COR, Inc. Lincoln NE, USA). All measurements were conducted on 10 cm^2^ areas of leaves attached to plants in leaf chambers with forced ventilation. The flow rate of gas was 9 cm^3^/s.

The leaflets were sampled after the measurements of photosynthetic rate for total chlorophyll assaying. The chlorophyll was extracted using 85% (v/v) acetone solution, and the contents were measured using a colorimetric method at 663 and 645 nm, respectively^15^.

### Measurements of starch and amino acid

Leaf samples for starch and amino acid determinations were taken at the 8 h into a photoperiod. Samples for starch assaying were extracted with 80% (v/v) ethanol, after discarding the extract, the residues were further extracted using 3% HCl solution, and then measured spectrophotometrically at 490 nm using phenol-sulphuric acid method^23^. Free amino acid was measured after 60% (v/v) methanol extraction using a mixture of ninhydrin in distilled water (2 g in 100 mL) and phosphate buffer solution (pH 6.6). Leaf extracts were added to the mix and heated at 85 °C for 25 min, and the absorbance was measured at 568 nm^24^. Leucine was used as internal standard.

### Measurements of carbon and nitrogen

All plants were divided into three organs types: roots, stalks (including branches) and leaves, and then dried (70 °C) and ground. Afterwards, samples were stored at room temperature until analysis. For measurement, 0.5 mg of samples were warped up with tin can (2 × 5 mm), and the total carbon and nitrogen concentration were determined by combusting in an element analyser (EA3000, Euro Vector, Italy).

### HPLC-DAD analysis of flavonoids

The fine ground samples for carbon and nitrogen measurements were used for flavonoids analysis. Samples were extracted using an ultrasonic-assisted method with 75% ethanol after removing the fat soluble impurities with petroleum ether. Total flavonoid content was determined using a colorimetric method with detection at 415 nm^25^. Flavonoid concentration in the extracts was calculated by referencing to a standard rutin (National Institute for the Control of Pharmaceutical and Biological Products, Beijing, China) curve (linearity range: 1.5–52.0 μg rutin/mL, R^2^ > 0.99) and expressed as milligram rutin equivalent per gram of dry weight (mg/g).

Individual flavonoids, quercetin, isoquercitrin and kaempferol, were determined using high performance liquid chromatograph (HPLC), and all extractions were filtered through a 0.45 μm polytetrafluoroethylene (PTFE) filter prior to HPLC analysis. Quercetin and kaempferol were quantified as aglycones after acid hydrolysis. An Agilent 1200 series system (Waldbronn, Germany) was used, which consisting of an online degasser, a quaternary pump solvent management system, an autosampler, a column heater, an UV/VIS diode array detector (DAD), and a data processing system. Quercetin and kaempferol were separated on an Eclipse Plus C18 column (250 mm × 4.6 mm, 5 μm) at 30 °C, and detected at 365 nm. The mobile phases were methanol (A) and 0.3% phosphoric acid (B) at 55: 45 (V_A_: V_B_). For isoquercitrin determination, the mobile phases were methanol (A) and 0.5% phosphoric acid (B). The gradient elution include 0–25 min, 15% A; 15–26 min, 15–90% A; 26–36 min, 90% A; 36–37 min, 90–15% A; and 37–45 min, 25% A. The detection wavelength was 350 nm. The standards quercetin, kaempferol (Sigma-Aldrich Inc., St. Louis, USA), and isoquercitrin (National Institute for the Control of Pharmaceutical and Biological Products, Beijing, China) were used to obtain an external calibration curve.

### Measurements of enzyme activity

To measure the levels of PAL, CHS and FHT in leaves of *C. paliurus*, Plant L-Phenylalanine ammonla-lyase (PAL) ELISA Kit, Plant chalcone synthase (CHS) ELISA Kit, and Plant Flavanone-3-hydroxylase (F3H) ELISA Kit were used, respectively. The purified plant PAL (or CHS, FHT) antibody was used to coat microtiter plate wells, followed by adding PAL to the wells, forming antibody-antigen-enzyme labelled antibody complex. After washing completely, TMB substrate solution was added for color developing at 37 °C for 15 min, and then the reaction was terminated with sulphuric acid solution and the absorbance was performed at 450 nm.

### Statistical analysis

For the analysis of variance (ANOVA), Duncan’s multiple-range test was used to calculate significant differences. All statistical analyses were performed at a 95% confidence level. Calculations were conducted using SPSS (version 16.0, SPSS Inc., Chicago, IL, USA).

## Results and Discussion

### Effects of N availability on photosynthetic rate and total chlorophyll content

**Figure 2:**
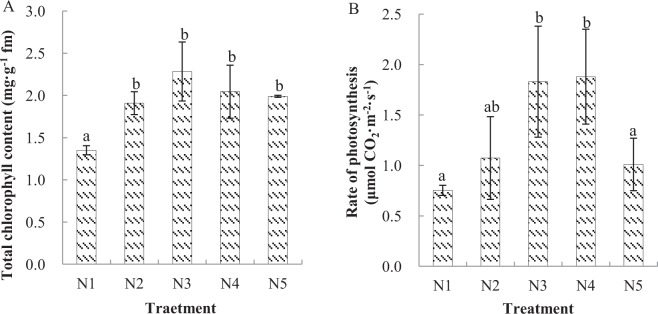
Effects of nitrogen treatments on total chlorophyll contents (**A**) and the photosynthetic rates (**B**) of *Cyclocarya paliurus* seedlings. ‘fm’ represents fresh matter. Different letters indicate significant differences among nitrogen treatments in the same category according to Duncan’s test (*p* < 0.05). N1, N2, N3, N4 and N5 represent the concentrations of NH_4_NO_3_ were 0, 0.19, 0.63, 1.13 and 2.00 mM, respectively.

The total chlorophyll contents in leaves of *C. paliurus* in the phytotron environment were relatively stable when subjected to treatments N2 (0.19 mM NH_4_NO_3_) through N5 (2.0 mM NH_4_NO_3_), while the contents decreased 41% under N-excluded conditions (Fig. 2A). The greatest net photosynthetic rates, which were significantly greater (*p* < 0.05) than those during treatments N1 and N5, occurred during treatments N3 and N4 (Fig. 2B).

Photosynthetic products are the energy bases of primary and secondary metabolisms. Therefore, primary and secondary metabolisms are tightly linked within plants through photosynthesis. Photosynthesis can be influenced by N availability because it affects N partitioning into photosynthetic pigments, enzymes, and the number and composition of chloroplasts^26^. A positive correlation exists between N availability and photosynthesis^27,28^, which corroborates our results from treatments N1 to N4. However, the photosynthetic rate during treatment N5 was significantly lower than during treatments N3 and N4. Under natural light conditions, the dry mass accumulation of *C. paliurus* linearly increases as the fertilization level increases, while the opposite pattern occurs under low light conditions^21^. Thus, the relative low light intensity conditions in phytotron may partially explain the photosynthetic rate pattern of the present study.

### C- and N- metabolisms were changed in response to N availability

**Figure 3:**
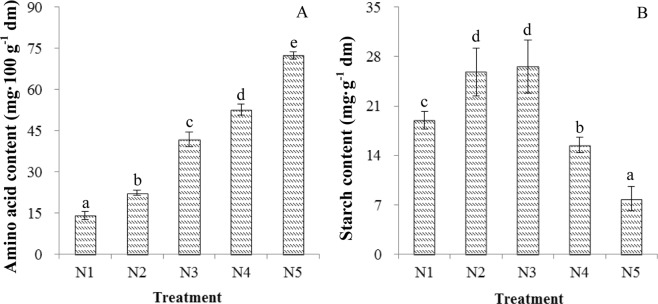
Effects of nitrogen treatments on free amino acid (**A**) and starch (**B**) level in leaves measured at 8 h into a photoperiod in *Cyclocarya paliurus* seedlings. ‘dm’ represents dry matter. Different letters indicate significant differences among nitrogen treatments in the same category according to Duncan’s test (*p* < 0.05). N1, N2, N3, N4 and N5 represent the concentrations of NH_4_NO_3_ were 0, 0.19, 0.63, 1.13 and 2.00 mM, respectively.

In the present studies, the total C levels in these three organs (leaf, stalk and root) were relatively stable under the five N-level conditions, while the total N levels linearly increased as the N fertilization increased, which resulted in a decreased internal C/N ratio (Table 1). Overall, the internal C:N balance were changed in response to N availability. Furthermore, N availability altered the N and C metabolic levels within plants. The levels of free amino acids linearly increased as the N availability increased (Fig. 3A), while the starch level presented a unimodal curve with the greatest amount occurring during treatment N3 (Fig. 3B). The variation patterns of starch and free amino acid levels indicated that N and C metabolic levels are tightly linked in almost every biochemical pathway within plants. This was confirmed by the total C and total N measurements in roots, stalks and leaves (Table 1).

**Table 1:** Variations in the carbon, nitrogen and carbon-to-nitrogen ratio (C/N) in the roots, stalks and leaves of *Cyclocarya paliurus* seedlings under five different nitrogen fertilization treatments.

Treatment	Root (%)^a^			Stalk (%)			Leaf (%)		
	nitrogen	carbon	C/N	nitrogen	carbon	C/N	nitrogen	carbon	C/N
N1	1.11a	42.31a	38.14c	0.62a	41.90a	68.05d	2.09a	42.48b	20.42c
N2	1.54ab	41.63a	27.28b	0.87ab	41.89a	48.33c	2.78b	43.24b	15.59b
N3	1.81bc	41.28a	23.36ab	1.07b	42.06a	40.58bc	2.72b	42.39b	15.59b
N4	2.17c	39.31a	18.16a	1.39c	41.55a	30.20a	2.85b	42.57b	14.95b
N5	2.11c	41.04a	19.91a	1.38c	42.22a	30.97ab	3.23c	39.22a	12.19a

^a^Different lowercase letters within a column indicate significant differences among nitrogen treatments in the same category according to Duncan’s test (*p* < 0.05). N1, N2, N3, N4 and N5 represent the concentrations of NH_4_NO_3_ were 0, 0.19, 0.63, 1.13 and 2.00 mM, respectively.

The C:N balance was influenced by N availability, as observed in other plants, such as grapevine, kale and tobacco^2,15,23^. The altered internal C:N balance could further affect plants on the following different levels: (1) gene expression involved in N assimilation, photosynthesis and secondary metabolism^8,14,15^; (2) the concentration and composition of metabolites^29,30^, which have important roles in determining the qualities of crops and medicinal plants; and (3) the root-to-shoot biomass allocation and morphology of plants^23,31^. In this study, the total C level was relatively stable, but the photosynthetic C allocation linearly increased with total N as the N availability level increased (Table 1), suggesting that a greater proportion of C was allocated to the N-assimilation pathway. This left less C for C-metabolite biosynthesis under high-N conditions, which was partially demonstrated by the variations in starch and amino acid levels (Fig. 3).

In high-N environments, the induced nitrate reductase activity and nitrate transport typically result in increased N assimilation. Therefore, the levels of N-assimilation (glutamic acid or glutamine) are considered indicators of a high internal N state^14^. Here, this was mainly evident at the amino acid level, and comparable results have been detected in other experimental systems^6,15^. Higher amino acid contents under high-N conditions imply that a large amount of C skeletons were allocated to N assimilation and the leaf carbohydrate content declined (Fig. 3B), which re-establishes the equilibrium between photosynthesis and C utilisation^15^. Starch is the main reserved carbohydrate in plants^11^, and it is an indicator of the resource pool available for allocation to different metabolic pathways. Primary metabolisms of plants are expected to receive priority for resources over secondary metabolisms. Thus, accumulation of secondary metabolites is usually occurred when there are abundant reserved carbohydrates within plants^9^, and the allocation trade-offs for C and N may occur among the biochemical pathways within plants.

Even though the C- and N-metabolic levels, which are regulated by the internal C:N balance, have been studied in response to phytochemicals or specific gene expression changes^14,15^, the mechanistic bases for the regulation remains to be revealed at the global gene express or transcriptional level.

### Effects of N availability on flavonoid accumulation and underlying correlation with the C:N balance

**Figure 4:**
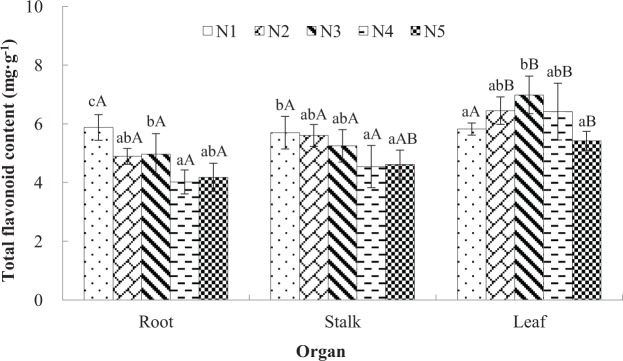
Effects of nitrogen treatments on the total flavonoid contents of different organs in *Cyclocarya paliurus* seedlings. Values within each graph followed by the different letters indicate significant differences among nitrogen treatments (lower case) and organs (upper case) (n = 3) according to Duncan’s test (*p* < 0.05). N1, N2, N3, N4 and N5 represent the concentrations of NH_4_NO_3_ were 0, 0.19, 0.63, 1.13 and 2.00 mM, respectively.

**Figure 5:**
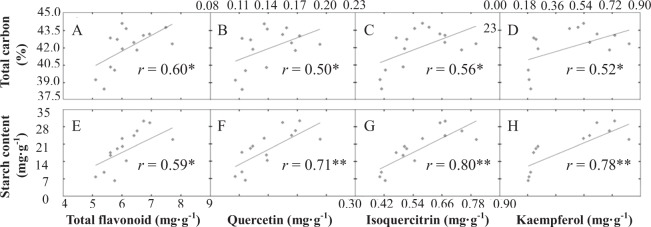
Correlations between the total carbon level and the contents of total flavonoid (**A**), quercetin (**B**), isoquercitrin (**C**), and kaempferol (**D**) in leaves of *Cyclocarya paliurus* after 15 d of five different N-level fertilization treatments. Correlations between the starch content and the contents of total flavonoid, quercetin, isoquercitrin, and kaempferol were marked (**E**–**H**), respectively.

As non-nitrogenous secondary metabolites, flavonoids were significantly influenced by N availability (*p* < 0.05). The greatest contents of total flavonoid were detected during treatment N1 in roots (5.88 mg/g dry mass (dm) and stalks (5.70 mg/g dm), being 28.9% and 19.1% more than under treatment N5, respectively (Fig. 4). However, flavonoid accumulation in leaves showed a different variation pattern. Intermediate N availability (N3) resulted in the greatest content (6.98 mg/g dm), and the lowest content was detected under treatments N1 (5.82 mg/g dm) and N5 (5.42 mg/g dm). Furthermore, three individual flavonoids were measured in this study. The main individual flavonoid was isoquercitrin, having an average content of 0.22 mg/g dm, followed by kaempferol (0.19 mg/g dm) and quercetin (0.06 mg/g dm) (Table 2). The variation patterns of the three individual flavonoids in different organs under the five N-fertilization conditions were similar to the patterns of total flavonoids. Of the organs evaluated, leaves had the greatest flavonoid content, while roots had the lowest flavonoid content. Along the N gradient, a significant positive correlation existed between flavonoids and the indicators of internal C status (total C and starch), while a stronger correlation existed between flavonoids and starch (Fig. 5A,B).

**Table 2:** Effects of nitrogen availability on the flavonoid contents of different organs in *Cyclocarya paliurus* seedlings.

Treatment	Individual flavonoid contents (mg/g)^a^								
	quercetin			isoquercitrin			kaempferol		
	root	stalk	leaf	root	stalk	leaf	root	stalk	leaf
N1	0.03dA	0.06 dB	0.12aC	0.06dA	0.12 dB	0.50bC	0.06dA	0.13 dB	0.23aC
N2	0.02cA	0.04cB	0.15bC	0.04cA	0.09cA	0.63cB	0.04cA	0.11cB	0.58cC
N3	0.02bA	0.03bA	0.18cB	0.04cA	0.07bA	0.74 dB	0.04bcA	0.08bA	0.76 dB
N4	0.01 aA	0.02 aA	0.15bB	0.03bA	0.04 aA	0.55bB	0.03abA	0.06 aA	0.46bB
N5	0.01 aA	0.02 aA	0.11aB	0.02 aA	0.04aB	0.41aC	0.03 aA	0.05aB	0.19aC

^a^Different letters indicate significant differences among nitrogen treatments (lower case, within column) and organs (upper case, within row) (n = 3) for the same category according to Duncan’s test (*p* < 0.05). N1, N2, N3, N4 and N5 represent the concentrations of NH_4_NO_3_ were 0, 0.19, 0.63, 1.13 and 2.00 mM, respectively.

*C. paliurus* is a relative fast-growing tree species, and it possesses high plasticity levels in anatomical structure, biochemistry and phenotype^21,32^. The biosynthesis and accumulation of flavonoids in *C. paliurus* leaves can be significantly induced by environmental factors, such as light intensity, soil nutrients and planting site^21,33^. Thus, the inducible secondary metabolites in this plant could be significantly influenced by fertilization, which has been partially demonstrated in a previous study^21^. However, limited information is available regarding the underlying regulatory effects of N fertilization. Understanding the regulatory effects of N on photosynthetic C allocation is important for the development of new agricultural practices that maintain high crop yields as well as the nutritional values of crops and medicinal plants.

The shikimate pathway, which links flavonoid biosynthesis and N metabolism within plants, catalyses the carbohydrates that come from the glycolysis and pentose phosphate pathways to synthesise aromatic amino acids (phenylalanine, tyrosine and tryptophan)^11^. The biosynthesis of flavonoids is catalysed by a series of enzymes, such as phenylalanine ammonia lyase (PAL), cinnamate 4-hydroxylase, 4-coumaroyl:CoA-ligase, chalcone synthase (CHS) and flavanone 3-hydroxylase (FHT). Lillo *et al*.^8^ revealed that the supply from the shikimate pathway is important for C flux into the flavonoid pathway, in which flavonoids are synthesised from phenylalanine. Many biotic and abiotic factors can trigger the activities of the above enzymes, such as nutrient limitation, light intensity and fungal infection^34–36^. Consequently, it is not surprising that flavonoid biosynthesis is regulated by N availability through photosynthetic C allocation among different biochemical pathways.

PAL, which functions at the intersection of primary and secondary metabolism and catalyses phenylalanine to form cinnamic acid, is frequently studied. Here, the PAL activities at low N levels (N1 and N2) were significant greater than at relatively high N levels (N3, N4 and N5) (Table 3). Kováčik and Klejdus^6^ also found that *Matricaria chamomilla* plants cultured under high N conditions had increased PAL activity levels and flavonoid contents in shoots. However, the greater PAL activity during low N availability did not result in a high flavonoid accumulation in *C. paliurus* leaves. This phenomenon was also observed in *Pyrus communis*^37^ and *Vitis vinifera*^38^. Furthermore, the CHS activity was significant greater at high N levels (N4 and N5), and its lowest activity level occurred in N3 (Table 3). Thus, the upstream genes in the flavonoid biosynthetic pathway (*PAL* and *CHS*) may not be key enzyme-encoding genes. Interestingly, the variation pattern of FHT activity was completely consistent with the flavonoid accumulation pattern in leaves of *C. paliurus*, which suggested that FHT is the key enzyme in the flavonoid biosynthetic pathway. *FHT* expression can be activated by carbohydrates and promote flavonoid accumulation^39^. Flavonoid and carbohydrate accumulations occurred under intermediate N levels, which indicated that N availability can impact the C:N balance and influence flavonoid accumulation within plants. Thus, the internal C:N balance can control not only the partitioning of C within the amino acid and carbohydrate biosynthesis pathways but also in the flavonoid biosynthesis pathway. However, more convincing work is needed to integrate biochemical and global gene expression changes related to C allocation between primary and secondary metabolisms in the future. In the present study, a strong positive correlation between flavonoids and reserved starch was evidenced by the Pearson’s correlation coefficients (*p* < 0.01, Fig. 5), which indicated that the C surplus within plants was a good indicator of the flavonoid accumulation. Indeed, plants experiencing intermediate to high N availability should have relative stable photosynthetic C, as shown by the variations in photosynthetic rate and total C (Fig. 2B and Table 1). Under the relative high-N conditions, a greater proportion of C was allocated to the N-assimilation, and the left less C was allocated to secondary metabolisms or reserve (Figs 3 and 4).

**Table 3:** Enzyme activities of PAL, CHS and FHT in leaves of *Cyclocarya paliurus*.

Treatment	Enzyme activity (OD_450_/g/min)^a^		
	PAL	CHS	FHT
N1	40.5c	40.3ab	179.2b
N2	45.2d	39.0ab	219.5c
N3	25.4ab	33.2a	372.3d
N4	25.6ab	42.8c	206.1b
N5	22.7a	51.6c	108.8a

^a^Different lowercase letters within a column indicate significant differences among nitrogen treatments in the same category according to Duncan’s test (*p* < 0.05). PAL, phenylalanine ammonia-lyase; CHS, chalcone synthase; FHT, flavonoid 3′-hydoxylase. N1, N2, N3, N4 and N5 represent the concentrations of NH_4_NO_3_ were 0, 0.19, 0.63, 1.13 and 2.00 mM, respectively.

The growth–differentiation balance hypothesis cannot be discounted based on the different variation patterns in response to N availability between leaf and stalk (or root) (Table 2). Saito^40^ showed that chloroplasts can participate in the primary synthesis of flavonoids. Additionally, *Arabidopsis* roots grown in complete darkness do not accumulate flavonoids because the expression levels of genes encoding flavonoid biosynthesis-related enzymes are light dependent^41^. Thus, leaves could be the sole tissue in which flavonoid biosynthesis occurs in higher plants. Buer *et al*.^42^ demonstrated that flavonoids can be transported long distances from their synthetic sites to distant tissues (root tips) through the vascular system. Therefore, the effects of N on flavonoid biosynthesis may be reflected only by the flavonoid accumulation in leaves (or in whole plants) rather than non-photosynthetic tissues. In addition, the long-distance movement of flavonoids has profound developmental effects, such as regulating auxin transport, controlling root branching and gravitropism^42^. The root-to-shoot biomass allocation induced by the C:N balance^21,22^ may partially explain the different patterns of flavonoid accumulations between leaf and stalk (or root) under the five different N treatments.

## Data Availability

The datasets generated during and/or analysed during the current study are available from the corresponding author on reasonable request.
